# Cyst Viability and Economic Significance of Hydatidosis in Southern Ethiopia

**DOI:** 10.1155/2019/2038628

**Published:** 2019-08-01

**Authors:** Getachew Gedeno Guduro, Angesom Hadush Desta

**Affiliations:** ^1^Samara University, College of Veterinary Medicine, P.O. Box 132, Samara, Ethiopia; ^2^Aksum University, Shire Campus, College of Agriculture, Department of Animal Science, P.O. Box 314, Shire, Ethiopia

## Abstract

Hydatidosis is cystic parasitosis caused by a larval stage of* Echinococcus granulosus *with immense economic and public health significance. A cross-sectional study was conducted from November 2016 to April 2017 in the city municipal abattoir of Southern Ethiopia with the aim of determining prevalence and cyst fertility and estimating financial losses associated with organ condemnation. The visceral organs of about 400 cattle were examined for hydatid cysts after slaughter. Postmortem examination, cyst characterization, and direct financial loss estimations were carried out. From the total 400 cattle examined, 208 (52%) were found positive for hydatid cyst infection in one or more of their organs. A total of about 395 hydatid cysts were collected from different organs of the infected cattle. Anatomical distribution of the cysts indicated that around 245 (62.03 %) were found in lung, 91 (23.04%) in liver, 26 (6.58%) in heart, 21 (5.32%) in spleen, and 12(3.04%) in kidney. From the total 395 cysts collected, 166 (42.03%) were found fertile and 229 (57.97%) nonfertile. From the total fertile cysts, 70 (42.17%) were found to be viable and 96 (57.83%) nonviable. Furthermore, from the total nonfertile cysts, 204 (89.08%) and 25 (10.91%) were sterile and calcified, respectively. Distribution of cyst calcification was higher in liver and fertility rate was higher in the cysts of lungs. The statistical analysis showed that the prevalence of hydatidosis was found to be significantly associated with age of the studied animals (P<0.05). However, there was no significant association (P>0.05) between the prevalence of bovine hydatidosis and other risk factors such as sex, breed, body condition, and origin of animals. The annual financial loss calculated from organ condemnation was estimated about 58,114.62 USD. This study revealed that hydatidosis is a highly prevalent disease in the study area with a huge economic losses. Therefore, there is a need for immediate intervention by breaking the life cycle of the parasite to alleviate its economic impact and zoonotic risks to humans.

## 1. Introduction

Ethiopia is naturally endowed with different agroecological zones and environmental conditions suitable for livestock production. The country is considered to have numerous livestock population in Africa. The country has about 54 million cattle, 25.5 million sheep, and 24.06 million goats by which majority of them are indigenous breeds [1]. The livestock sector is an important means of livelihoods of many Ethiopian farmers and has a significant contribution to the national economy. It has a contribution of around 16.5% to the national Gross Domestic Product (GDP) and about 35.6% to the agricultural GDP [2]. In addition, it is a means of 15% national annual export earnings and 30% agricultural employment and sustains livelihood of 80% of all rural population [3].

Though the country has high livestock population and favorable environmental conditions, the benefit from livestock output is insignificant. The factors causing this low productivity are more complex and interrelated including inadequate feed and nutrition, traditional management system, and widespread diseases [4]. Among many prevalent livestock diseases, parasitosis represents a major health problem hampering livestock productivity in tropics including Ethiopia [5]. Hydatidosis is among the major parasitic diseases that has reduced meat production due to carcass or organ condemnation in abattoirs [6]. It is a cystic echinococcosis caused by the larval stage (metacestode) of* Echinococcus granulosus *with a great economic and public health significance. As to many other parasitic infections, the life cycle of* Echinococcus *infection is complex which requires two mammalian hosts (intermediate and definitive hosts) for the completion of its life cycle [7].

The usual definitive hosts for this parasite are dogs and many mammalian species including domestic ungulates and man can be intermediate hosts [8]. The gravid proglottids of the adult worm which resides in the small intestine release eggs that are passed in the feces of the definitive host. These infective eggs with oncosphere can be accidentally ingested by an intermediate hosts such as cattle, sheep, goats, pigs, other ruminants, and humans. Then hatching of the eggs will occur in the small intestine of the intermediate host, and the released oncosphere will penetrate the intestinal wall and reaches different organs, in particular the liver and lungs through the circulatory system. Therefore, life cycle of hydatidosis is completed after fertile cyst is eaten by the definitive host [9].

The disease has worldwide occurrence, and it can cause considerable economic losses and public health problems in many countries. This is due to the tape worm has an ability to adapt to a wide variety of domestic and wild intermediate hosts [10]. Hydatidosis causes decreased livestock production and condemnation of offal's containing the cysts in slaughter houses [11]. It is more prevalent in developing countries where the dog lives in close relationship with man and domestic herbivores and feeding on infected offal [12, 13].

In Ethiopia, hydatidosis is known and documented since the 1970s and it is one of the major causes of organ condemnation in most abattoirs and slaughter houses and leads to huge economic losses [14–16]. It is potentially a zoonotic parasite in areas where cattle, sheep, and goats are still slaughtered traditionally and offal is easily accessible to scavenging dogs and other wild carnivores. Furthermore, absence of proper meat inspection, poor management of food animals, traditional practices of backyard farming system, lack of sufficient awareness about food borne diseases, and presence of large stray dog population are thought to contribute significantly to the prevalence of the disease in Ethiopia [17].

There are human cases of hydatidosis that are frequently reported from various areas of the country most commonly in the rural areas of Ethiopia where dogs and domestic animals live very closely. The disease is more dangerous in human than in domestic animals [18]. It is one of the economically important parasites in man and farm animals [19]. Hence, there is a need to investigate the viability of the cysts that can play in transmission of the parasite, and the economic significance by estimating the financial loss occurred due to organ condemnations by this parasite. Therefore, the objectives of this study were to determine the prevalence, cyst characterization, and associated risk factors for transmission and economic significance of the disease.

## 2. Material and Methods

### 2.1. Description of Study Area

The study was conducted at municipal abattoir found in the capital city of Southern Ethiopia. Hawassa is the capital city of Sidama zone administration and Southern Nations Nationalities and Peoples' Regional State and it is 275 km South of Addis Ababa. It geographically lies between 4°27′ and 8°30′ N latitude and 34°21′ and 39°1′ E Longitude at an altitude of 1,790 m above sea level. The area receives average rain fall annually ranging from 800 to 1,000 mm of which 67% falls in the long rainy season, which extends from June to September. During the study period, the mean minimum and maximum temperatures of the area were 20.1°C and 30°C, respectively, and 51.8% mean relative humidity. The area is mainly covered by dry savannah and bush type of vegetation [20].

Based on the 2007 census conducted by the CSA, this zone has a total human population of 2,954,136, of whom 1,491,248 are men and 1,462,888 women, with an area of 6, 538.17 square kilometers. The total livestock population of Sidama zone is estimated at 1,721,341 cattle, 228,941 goats, 457,465 sheep, 57,643 horses, 54,066 donkeys, 725,540 poultry, and 44,492 bee hives [21].

### 2.2. Study Population

The study animals were comprised of both cross and indigenous cattle breeds of both sexes brought for slaughter at the municipal abattoir. But majority of the animals brought for slaughter were zebu cattle and very few were cross breeds. It was difficult to accurately indicate the origin of all animals slaughtered at the municipal abattoir and to relate it with the findings on hydatidosis to a particular locality. However, certain attempts made in this regard revealed that majority of them were brought from nearby markets (Shashemene, Arsi-Negelle, Wolayta Sodo, Borana, Tikurwoha, and Tula).

### 2.3. Study Design

A cross-sectional study was conducted from November 2016 to April 2017. Both antemortem and postmortem inspection procedures were carried out during the study periods.

### 2.4. Sampling Methods

Simple random sampling technique was employed in the lairage to select the required number of study population. Prior to sampling, each selected animal was given an identification number and data on each animal's sex, age, breed, and origin was recorded. During meat inspection, identified animals and their respective organs were strictly examined separately to avoid mixing up of organs. Meat inspection was made in accordance with the procedures of Ethiopian Ministry of Agriculture Meat Inspection Regulation (1972) for the detection of hydatid cysts. Visual inspection was done followed by multiple incisions of 0.5 cm in each organ.

### 2.5. Sample Size Determination

The sample size was determined by considering previous prevalence of 52.69 % [6] with 95% confidence interval and 5% absolute precision.

The formula used to calculate the sample size is shown below [22],

(1) $$\begin{array}{c} N=\frac{{\left(1.96\right)}^{2}Pexp\left(1\text{-}Pexp\right)}{d^{2}} \end{array}$$

where Pexp is expected prevalence (52.69 %),

N is required sample size,

d is desired absolute precision.

Therefore, according to this formula the calculated sample size was determined to be 383. But with the intention to increase the level of accuracy, a total of 400 cattle were included in the study.

### 2.6. Study Methods and Data Collection

#### 2.6.1. Antemortem Examination

Antemortem inspection recommended [23] was applied. Information on the sex, age, and body condition of each animal was recorded. Based on the body condition, animals were grouped as good and medium [24]. The age of each animal was estimated on the basis of dentitions [25] and in this regard only two age groups were considered; adult (7 to 9 years) and old (≥ 10 years). Young age group is excluded from the study due to reasons that most local farmers do not sell their cattle at young age but most commonly sell after many years of providing draft power. Moreover, the abattoir is not an export abattoir, which mostly focuses on matured ages.

#### 2.6.2. Postmortem Examination

Postmortem inspection was carried out according to the procedures recommended by food and agricultural organization [26]. Visceral organs, particularly lung, liver, heart, spleen, and kidney, were carefully inspected with visualization, palpation, and incision. Cyst containing organs from infected animals were collected and number of cysts per organ was recorded on the data collection format sheet prepared for this purpose.

### 2.7. Characterization of Hydatid Cysts

Cysts were collected and transported to laboratory for characterization. Gross examination of individual cyst for any evidence of degeneration and calcification was made. Fertility and viability test was also conducted on the collected cysts. Individual hydatid cysts were collected and the cyst wall was carefully incised and the contents were poured in to a clean glass Petri dish and examined under a microscope (40×) for the presence of hydatid protoscolices, which if present, seen as white dots on the germinal epithelium or brood capsule or hydatid sands within the suspension; such cysts were characterized as fertile cysts. Then the fertile cysts were further subjected to viability test. A drop of the sediment containing the protoscolices were placed on the microscope glass slide and a drop of 0.1% aqueous eosin solution was added to equal volume of hydatid fluid on the microscope slide and then covered with cover slip and observed under microscope (40×) with the principle that viable protoscolices should completely or partially exclude the dye while the dead (nonviable) ones absorb it [27]. Furthermore, infertile cysts were further classified as sterile or calcified. Sterile hydatid cysts were characterized by their smooth inner lining usually with slightly turbid fluid in their content. Typical calcified cysts produce a gritty-sound heard at incision [28].

### 2.8. Assessment of Financial Loss Associated with Hydatidosis

In assessing the economic loss of the disease, only direct financial loss was considered. Direct losses were calculated on the basis of condemned organs. For calculating the cost of condemned organs four retailers in the town were interviewed randomly to establish the local market price per unit organ. Then average price was drawn out from the data and used to calculate the loss accordingly. Annual financial loss of the disease was then calculated based on total number of organs condemned, average price per organ, and average annual slaughter rate of the abattoir. The average annual slaughter rate was calculated from the past 3 years slaughter rate data record of the abattoir in which 8200 slaughter rate in 2014, 9000 slaughter rate in 2015, and 10,000 slaughter rate in 2016 were considered. The financial loss due to organ condemnation was then calculated by using the formula described as follows [6]:

(2) LOC=NAS×Ph×Plu×Cplu+NAS×Ph×Phr×Cphr+NAS×Ph×Pli×Cpli+NAS×Ph×Psp×Cpsp+NAS×Ph×Pkid×Cpkid

where LOC is loss due to organ condemnation;
  NAS is mean number of cattle slaughtered annually;
  Ph is prevalence of hydatidosis;
  Plu is percent involvement of lung;
  Cplu is current mean retail price of lung;
  Pli is percent involvement of liver;
  Cpli is current mean retail price of liver;
  Phr is percent involvement of heart;
  Cphr is current mean retail price of heart;
  Psp is percent involvement of spleen;
  Cpsp is current mean retail of spleen;
  Pkid is percent involvement of kidney;
  Cpkid is current mean retail of kidney.

### 2.9. Data Management and Analysis

The data obtained from antemortem and postmortem findings in the abattoir was coded in Microsoft excel sheet and subjected to descriptive statistics and chi-square test in order to assess the magnitude of the difference of comparable variables using SPSS version 20 software of computer program. Statistically significant association between variables was considered to exist if the p-value is less than 0.05.

## 3. Result

This study showed that, from a total of 400 cattle slaughtered at the municipal abattoir, 208 (52%) cattle were found harboring at least one hydatid cyst in their different organs. From the positive cases, there were total of 21 multiple organ infection cases and the rest involved only single organ.

The direct economic loss due to organ condemnation from the inspected total sampled cattle slaughtered in Hawassa municipal abattoir was calculated to be 413.20 USD (Table 1).

The average annual financial loss due to organ condemnation as a result of hydatidosis was assessed based on the total number of organs condemned, average price per organ, and average annual slaughter rate of the abattoir in the past three years from 2014 to 2016. Therefore, the calculated average annual financial loss of the municipal abattoir due to hydatidosis was estimated to be 58,114.62USD.

During the study, a total of 21 multiple organ infection was found and 15 of them were lung and liver (Table 2).

During the cyst characterization tests, both fertile and nonfertile cysts were found except in kidney where only sterile cysts were found (Table 3)

According to the chi- square test, it was only age (P≤0.001) that showed highly significant association with the occurrence of hydatidosis in Hawassa municipal abattoir (Table 4).

## 4. Discussion

Hydatidosis is known to be an important disease in livestock and human in different parts of the world and its prevalence and economic significance has been reported by different researchers in different geographical areas. The current study revealed that the overall prevalence of bovine hydatidosis at the municipal abattoir was found to be 52%. This finding is in agreement with the findings who reported a prevalence of 52.69 % in Ethiopia [6]. But the current result is higher than the findings reported in other parts of the country such as 16% in WolaitaSodo [29], 22% in Tigray region[14], 48.9% in Debre Markos [30], 32.1% in Mekelle [31], 17.5% in Wollo [32], 40.5% in Addis Ababa [33], 33.5% in Kombolcha [34], and higher than some findings in other countries like 48.7% in Tanzania [35] and 2.8% in Sudan [36]. However, the current prevalence is lower than the one reported in Assela which was 61% [37].

Generally the variation in prevalence among different geographical locations could be ascribed to the strain differences of* Echinococcus granulosus *that exist in different geographical locations. Other factors like difference in culture, social activities, animal husbandry systems, and attitudes to dogs in different regions may contribute to variation [12]. The prevalence of 52% in the study area was high. This may be attributed to the abundance and frequent contact between the infected intermediate and final hosts. It could also be related to slaughtering of aged cattle which have had considerable chance of exposure to the parasitic ova, backyard slaughtering of small ruminants and provision of infected offal's to pet animals around homesteads. Additionally, lack of proper public awareness about the disease and presence of few slaughter houses could have contributed to such a high prevalence rate.

Attempts made to assess the relation between the infection rate and sex of the animals revealed that no significant association exists between sex and occurrence of the disease [38]. This may be due to indiscriminate exposure to risk irrespective of sex in the management of the study area. In reverse, it disagrees with the research findings who reported as the disease had occurred more in females than males [39]. The explanation behind this was female animals are not slaughtered in younger ages as long as they are fertile. Female animals are sent to abattoir after milking and getting calves for years. In regard to the rate of infection of hydatidosis in different age groups of cattle, significant difference (P<0.005) was observed. The study similar with other findings indicated that old animals were highly infected [6, 40, 41]. This could be mainly due to their longer exposure to* E. granulosus *and to lower immunity against the infection. In the present study, breed of cattle was not showed significant association with prevalence of hydatidosis [42].

Statistical analysis was also made to establish relationship between animal origin and prevalence of the disease and it was not significantly related (P>0.05) [6]. This may be due to difficulty in getting exact origin of the animals and exchanging of the animals in local markets. But this finding contradicts with who reported that there is significant correlation between bovine hydatidosis and animal origin [43] that could be attributed to difference in social activity, attitudes to dogs, and climatic conditions in different regions.

Analysis was also made to find a relation of the disease with body condition score of the animals, but the result showed no statistically significant association to exist between the disease and animal body condition score [6]. With this regard, it contradicts with the study conducted [44] who reported that animals with poor body condition are highly infected followed by medium and good.

In terms of organ distribution of hydatid cyst, this study indicated that lung and liver were the dominant organs affected, with prevalence of 62.03% and 23.04%, respectively. This result is in agreement with the findings of [45–47]. The fact that hydatid cysts showed greater preference for lung and liver than other viscera could be related to presence of the dense capillary networks in these organs which filter out and retain the oncosphere of* Echinococcus granulosus* before being encountered by peripheral organs. The higher prevalence in lung might also be associated with the fact that cattle are slaughtered at older age. At this period, the capillaries of liver are dilated and most cysts passed to the lungs. Besides this, it is possible for the hexacanth embryo to enter the lymphatic circulation and be carried via the thoracic duct to the heart and lung in such case the lung will be infected before liver [48].

In examination of the fertility and viability condition of the cyst, around 51.65% was sterile, 42.03% fertile, and 6.33% calcified cysts. In comparison with the fertility rate among the organs, it was higher in lungs than liver. The explanation behind this was that the relatively softer consistency of lung tissue allows easier development of the cysts and favors their fertility rate [15]. High percentage of cyst calcification was found in the liver than in the lungs. This may be associated with the relatively higher reticuloendothelial cells and abundant connective tissue reaction of the organ which encapsulates the cyst with in a fibrous wall up to 13 mm thickness [6].

In this study significant financial loss was calculated due to hydatidosis with an estimated loss of 58114.62 USD per annum. Different financial losses regarding bovine hydatidosis were also reported from different parts of the country [14, 15, 49]. The difference in the amount of economic loss could be due to the variation in the prevalence of the disease, retail market price of organs, and mean annual slaughter rate in different abattoirs.

## 5. Conclusion

This study demonstrated that hydatidosis/echinococcosis is highly prevalent parasitic disease which is imposing serious economic losses due to organ condemnation in the city municipal abattoir. This may be attributed to the presence of large stray dog population which play significant role in the transmission of the disease. Moreover, factors such as habit of feeding affected offal to pet animals, lack of public awareness about the disease, lack of infrastructures and services to carry out proper meat inspection at all levels, and traditional animal husbandry systems have contributed to disease occurrence. The statistical analysis of risk factors with respect to occurrence of the disease indicated that age of the animal is significantly associated. During this study, lung and liver were the mainly affected and frequently condemned organs that have caused significant financial loss in the study abattoir. A relatively higher calculated annual financial loss due to organ condemnation because of this parasite is recorded. Therefore, backyard slaughtering practices and provision of affected offal to pet animals should be avoided. Stray dogs controlling, domestic ruminant deworming should be regularly conducted. Furthermore, establishing well-equipped and standardized municipal abattoirs and proper meat inspection should be carried out. Awareness about public health implication and economic importance of the disease should be created to the community.

## Figures and Tables

**Table 1:** Direct economic losses associated with hydatidosis from inspected sample cattle slaughtered at Hawassa municipal abattoir (N=400).

Organ	Number of organs condemned	Price per organ	Total price
		(USD)	(USD)
Lung	118	1.00	118
Liver	63	4.00	252
Heart	21	1.20	25.20
Spleen	17	0.80	13.60
Kidney	11	0.40	4.40
Total	230	-	413.20

**Table 2:** Multiple organ infection of hydatidosis from sample taken at Hawassa municipal abattoir.

Organ	No of infected cases	Relative percentage	Total cyst
Lung and liver	15	71.43%	55
Lung and spleen	3	14.29%	7
Lung and heart	1	4.76%	3
Lung and kidney	2	9.52%	4
*Total*	21	100%	69

**Table 3:** Characterization of hydatid cysts in different organs of affected cattle.

Organ	Fertile cysts		Non fertile cysts		Total (%)
	Viable (%)	Non-viable (%)	Sterile (%)	Calcified (%)	
Lung	61(24.90 )	58 (23.67 )	120 (48.98 )	6 (2.45)	245 (62.02)
Liver	9 (9.89 )	22 (24.18 )	44 (48.35 )	16 (17.58 )	91 (23.04)
Heart	0	7 (26.92 )	16 (61.54 )	3 (11.54 )	26 (6.58)
Spleen	0	9 (42.86 )	12 (57.14 )	0	21 (5.32)
Kidney	0	0	12 (100 )	0	12 (3.04)
*Total*	70 (17.72)	96 (24.30)	204 (51.64)	25 (6.34)	*395* (100)

**Table 4:** Chi-square analysis of risk factors with prevalence of hydatidosis.

Risk factors	Category	No of animals examined	No of positive animals (%)	X^2^- value	P- value
Sex	Male	332	172(51.81)	0.29	0.865
	Female	68	36(52.94)		
Age	Adult	254	101(39.76)	41.744	≤0.001
	Old	146	107(73.29)		
Breed	Local	383	200(52.22)	0.174	0.677
	Cross	17	8(47.06)		
Body condition	Good	291	143(49.14)	3.497	0.61
	Medium	109	65(59.63)		
Origin	Arsinegelle	79	44(55.69)	6.213	0.286
	Tula	85	44(51.76)		
	Tikurwoha	58	32(55.17)		
	WolaitaSodo	56	21(37.5)		
	Shahamane	30	18(60)		
	Borana	92	49(53.26)		
*Total*		400	208 (52.00)		

## Data Availability

The data used to support the findings of this study are included within the article.
